# Production of Motor Gasoline Components from Plastic Waste by Pyrolysis Followed by Hydrosaturation of Fuel Fractions

**DOI:** 10.3390/polym18131564

**Published:** 2026-06-23

**Authors:** Andrey Altynov, Daniil Eronskiy, Maria Kirgina, Kirill Larionov, Ilya Bogdanov

**Affiliations:** 1 Research School of Chemistry & Applied Biomedical Sciences, Tomsk Polytechnic University, 30 Lenina Avenue, 634050 Tomsk, Russia; andrey_altynov@tpu.ru; 2 School of Earth Sciences & Engineering, Tomsk Polytechnic University, 30 Lenina Avenue, 634050 Tomsk, Russia; 3 School of Energy & Power Engineering, Tomsk Polytechnic University, 30 Lenina Avenue, 634050 Tomsk, Russia; larryk@tpu.ru@tpu.ru

**Keywords:** pyrolysis, gasoline, plastic waste, hydrogenation, catalyst

## Abstract

In the context of a constantly deteriorating environmental situation, in particular due to the uncontrolled accumulation of plastic waste, the search for effective ways to recycle plastic is an urgent task. Pyrolysis of plastic waste followed by the hydrosaturation of liquid products may become a promising method for obtaining components of motor gasoline. The aim of this study is to obtain motor gasoline components from plastic waste through pyrolysis, followed by hydrogenation of the fuel fractions for their use in the production of commercial fuels. The scientific novelty of this study consists of establishing the influence of hydrosaturation process parameters on an Al-Co-Mo hydrotreating catalyst (temperature and feedstock flow rate) on the transformation of hydrocarbons present in the gasoline fraction separated from the liquid pyrolysis products of polypropylene waste. The most preferred conditions for obtaining feedstock for subsequent hydrosaturation of polypropylene waste turned out to be the pyrolysis process carried out at a temperature of 450 °C and atmospheric pressure. Based on calculations in the Compounding software, promising blending components were identified. Based on the obtained results, two samples were identified as having the greatest potential for blending commercial gasolines in terms of hydrocarbon composition and performance characteristics. The sample obtained at the hydrosaturation process parameters of 350 °C and a feedstock flow rate of 0.51 mL/min is the most preferable in terms of its composition, since it demonstrates a minimal content of olefins (18.7% vol.) and benzene (0.87% vol.) but has a relatively low octane number (RON 58.7). The sample obtained at the hydrosaturation process parameters of 300 °C and a feedstock flow rate of 0.85 mL/min has relatively higher octane characteristics (RON 72.9) and can be used as a high-octane component but requires blending with components that compensate for the increased olefin content. Also, it is shown in this work that hydrosaturation of the gasoline fraction separated from the liquid pyrolysis products of polypropylene waste enables the production of motor gasoline components whose blending rate in commercial gasolines recipes can reach up to 35% by volume.

## 1. Introduction

One of the most significant challenges of the 21st century is the environmental situation, particularly the issue of recycling human waste, including plastic waste, which, due to improper disposal and uncontrolled accumulation, has a negative impact on the environment and human health.

Furthermore, over the past several decades, energy consumption has been on the rise, particularly with the consumption of hydrocarbon feedstocks, from which the most widely used product is motor fuels.

It is important to note that the feedstock for both plastic and fuel production is fossil hydrocarbons, and despite obvious differences in the structure and properties of these products, both are hydrocarbons.

Therefore, it is crucial to research and develop processes for recycling waste plastic into useful products such as motor fuel components. This would simultaneously address both issues of effectively recycling plastic waste and expanding the feedstock pool for fuel production, which is especially important given the depletion of oil reserves.

According to [1,2], by 2023, more than 400 million tons of plastic were produced globally, more than 300 million tons of which became waste. Approximately 30% of this plastic waste was incinerated, resulting in the release of toxic substances into the atmosphere [3], approximately 45% was landfilled, which effectively represents a loss of valuable and already extracted hydrocarbon resources [4], and more than 10% was improperly disposed of, becoming microplastics [4,5,6,7].

Incorrectly recycled plastic waste, turning into microparticles, can accumulate in water, food, and air. As a result, microplastic particles enter the human body and accumulate there. This leads to the development of serious diseases [8,9,10,11].

Another significant problem of recent decades is the growth in the consumption of hydrocarbon energy sources [12], in particular liquid motor fuels. According to Rosstat data for 2023, in Russia alone, the number of passenger cars per 1000 people was 327 units, i.e., every third Russian owns a passenger car [13]. At the same time, news constantly appears that hydrocarbon reserves are not endless, in particular oil, as the world is supplied with it for only 50 years [14]. Meanwhile, demand for oil and petroleum products is growing every year; by 2026, the IEA predicts an increase to 104.79 million barrels per day [15].

As part of this area of research, several strategies for the efficient utilization of plastic waste and its conversion into valuable fuel and materials have been considered [16,17,18]. Chemical and thermal degradation are recognized as the most effective. Using thermal processes, plastic waste may be a potential source of hydrocarbon feedstock for producing valuable products such as hydrocarbon gases, components of liquid motor fuels, feedstock for petrochemical production, etc. [19,20,21]. This approach not only allows for the disposal of hazardous waste, which, among other things, occupies significant areas, but also allows for the production of valuable products for various industries. One possible technological process for producing liquid hydrocarbons from plastic waste is pyrolysis (pyrolysis oil) [22,23]. The main problems with these thermal processes are high energy consumption, the low thermal conductivity of plastics, and the low quality of the resulting products [23,24]. The products obtained through these processes are complex mixtures of hydrocarbons with a wide boiling range, which allows them to be considered as components of motor fuels [25,26,27]. Thus, a number of studies have demonstrated the use of pyrolysis oils for blending with diesel oil fractions [28,29,30,31], which is possible because the content of olefinic hydrocarbons in diesel fuels is not limited by standards. At the same time, in the products of plastic pyrolysis exist largely unsaturated hydrocarbons, the content of which in commercial motor gasolines is strictly regulated. This fact limits the use of hydrocarbon fractions obtained by plastic pyrolysis in the production of commercial motor gasolines. A possible solution to this problem is the hydrogenation (hydrosaturation) of the fractions obtained during plastic pyrolysis, which will effectively remove the restriction on their use in the production of commercial fuel products. Today, some works are already considering the process of hydrogenation of pyrolysis oil in order to study the composition and conversion of olefins and diolefins [32]; there are also works on studying the hydrorefining of pyrolysis oil in order to obtain jet fuel [33,34], and some works are also devoted to the hydrogenation of pyrolysis products of bio-feedstock [35,36,37,38].

This work is devoted to identifying the patterns of influence of technological parameters of the process of hydrosaturation of the gasoline fraction isolated from the products of pyrolysis of plastic waste on the composition and characteristics of the obtained products, as well as assessing the feasibility of involving the obtained products in the blending of commercial gasolines.

The results obtained in this study provide the foundation needed to develop industrial processes for recycling polymer waste to produce motor gasoline components. Obtaining data on the relationship between the composition, mode, product distribution, and distillate properties will partially bridge the gap between empirical observations and engineering calculations, enabling targeted increases in the utilization of low-boiling fractions obtained by plastic pyrolysis in the production of commercial fuels.

## 2. Materials and Methods

The objects of this study were samples of liquid polymer waste pyrolysis products (pyrolysis oil), a gasoline fraction separated from the pyrolysis oil, and its hydrosaturation products on an Al-Co-Mo hydrotreating catalyst.

The study focused on the composition and characteristics of the feedstock and catalytic hydrosaturation products, as well as the patterns of chemical transformations of the hydrocarbons present in the gasoline fraction separated from the pyrolysis oil.

The following physicochemical and performance characteristics were determined for the resulting pyrolysis oil: density, kinematic viscosity, and fractional and group compositions. The hydrocarbon composition of the separated gasoline fraction and its catalytic hydrosaturation products was determined. Furthermore, based on the hydrocarbon composition data for the separated gasoline fraction and its catalytic hydrosaturation products, the physicochemical and performance characteristics were calculated using the «Compounding» modeling system.

In this work, the physicochemical and operational characteristics, as well as the composition of the studied samples, were determined using the following methods and equipment:

1. Kinematic viscosity at 20 °C was determined using a calibrated glass capillary viscometer. Tests were conducted in accordance with the procedures presented in [39].
2. Density of samples at 20 °C was determined using a pycnometer. Tests were conducted in accordance with the procedures presented in [40].
3. Hydrocarbon composition of the gasoline fraction separated from pyrolysis oil and the resulting hydrosaturation products was determined by gas chromatography on a Chromatec-Crystal 5000 (Chromatec, Yoshkar-Ola, Republic of Mari El, Russia) unit with a 25 m × 0.22 mm quartz capillary column, stationary phase—SE-54, carrier gas—helium; tests were conducted in accordance with the procedure presented in [41].
4. The fractional composition was determined, and the gasoline fraction was separated from the pyrolysis oil using an ARN-LAB-03 unit for distilling oil and petroleum products (JSC «LOIP» St. Petersburg, Russia), according to the method presented in [42].
5. The group composition of the obtained pyrolysis oil samples was determined at various temperatures using chromatograph mass spectrometry on a Chromatec Crystal 5000.2 unit (Chromatec, Yoshkar-Ola, Republic of Mari El, Russia) with an HP-1-MS column (30 m; 0.25 mm; 0.25 μm) (Santa Clara, CA, USA).
6. The «Compounding» modeling system was used to determine such characteristics of hydrosaturated gasoline fractions as research octane number (RON), motor octane number (MON), saturated vapor pressure (SVP), density, and volumetric hydrocarbon composition [43].

## 3. Results

### 3.1. Methodology for Pyrolysis of Polymer Waste on a Laboratory Unit

**Figure 1:**
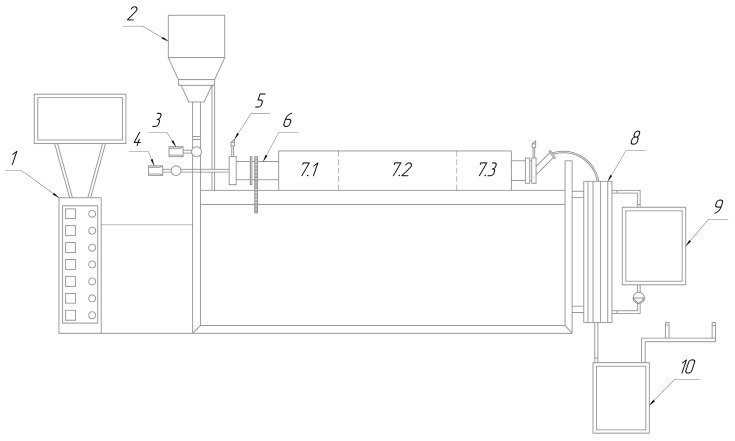
Schematic diagram of a laboratory-scale polymer waste pyrolysis unit: 1—reactor control unit; 2—receiving bin; 3—dosing drive; 4—drive; 5—thermocouple; 6—reactor tube; 7.1, 7.2, 7.3—combined reaction zones of the pyrolysis reactor; 8—condenser; 9—cooler; 10—finished product receiving bin.

A laboratory unit (Figure 1) was used to pyrolyze polypropylene waste. Particles obtained by grinding household polypropylene waste from a landfill were used as feedstock. To obtain feedstock particles, the plastic was crushed to a particle size of 3–4 mm. The products of the process are hydrocarbon gases, liquid pyrolysis products (pyrolysis oil), and a solid residue. Depending on the temperature in the reactor, the yield of products in the pyrolysis process is: liquid compounds 68.9–93.0% by weight, gaseous 6–31% by weight, and solid residue 0.1–1% by weight.

The process of polypropylene waste pyrolysis was implemented on a laboratory unit, which is designed to study the processes of thermal decomposition of polymers at atmospheric pressure without air access. Feedstock are fed into a receiving bin (2) with a dispenser (3). The dispenser is connected by a pipeline to the housing of the screw conveyor (4). Through the pipeline, the feedstock from the dispenser is fed into the housing of the screw conveyor, from where it is are fed by a screw into a drum-type reactor (7), passing through an intermediate tank (5). After the reactor, the gas-phase products formed during the pyrolysis process enter the condenser (8), where they condense and are directed through a pipeline to the liquid hydrocarbon accumulation tank (10). At the top, the condenser is connected to a tank (9) filled with water. The temperature of the cooling water is maintained at 15 °C. The liquid hydrocarbon accumulation tank is equipped with a branch pipe for the discharge and analysis of obtained gas. The system also has a sampler for non-condensable gas-phase compounds of pyrolysis, which is installed after the condenser. The reactor parameters are controlled using a remote unit and an automated workstation (1).

Table 1 presents the process parameters under which the pyrolysis process of polypropylene waste was carried out.

### 3.2. Methodology for Hydrosaturation on a Laboratory Unit

A gasoline fraction with a boiling point up to 180 °C was separated from the polypropylene waste pyrolysis products obtained under various process parameters by fractionation.

**Figure 2:**
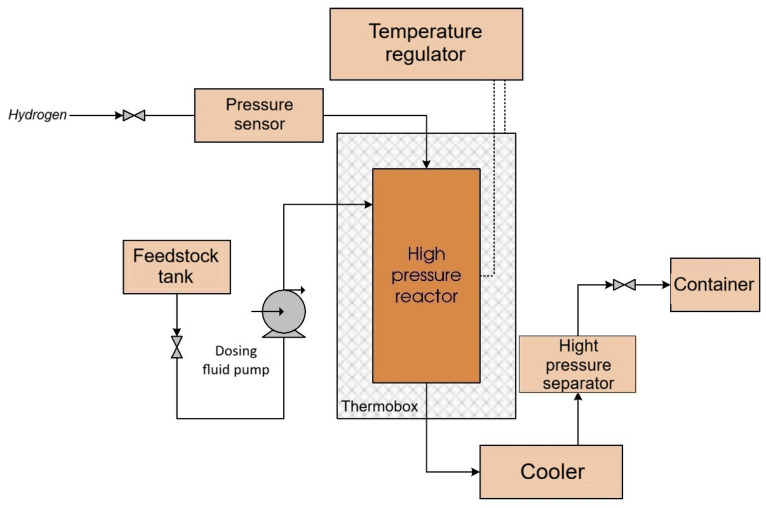
Block diagram of the catalytic unit.

Hydrosaturation of the gasoline fraction separated from the pyrolysis oil was carried out using a laboratory catalytic unit (Figure 2), which is designed to study processes occurring under elevated pressure in a flow reactor at a maximum pressure of 90 bar and a maximum temperature of 700 °C.

During the process, the feedstock is fed by a piston liquid metering pump into a high-pressure reactor located in a thermal box. The resulting product is then sent to a water condenser and then to a high-pressure separator, where light hydrocarbon gases are separated (product stabilization occurs). After stabilization, the liquid product enters a receiving tank. Process parameters are monitored using pressure sensors and a multichannel microprocessor-based temperature controller.

A 10 cm^3^ catalyst was placed in a fixed-bed reactor with an internal diameter of 12 mm.

The commercial industrial complex Al-Co-Mo catalyst was used as the hydrosaturation catalyst, and its detailed characteristics are presented in Table 2.

The technological parameters for the catalytic hydrosaturation process and sample coding are presented in Table 3.

## 4. Discussion

### 4.1. Results of Determining the Characteristics and Composition of Polypropylene Waste Pyrolysis Products

To separate the gasoline fraction from the pyrolysis oil and subsequently implement its hydrosaturation, the first step involved determining the physicochemical characteristics of the pyrolysis oils obtained under various process parameters of the polypropylene waste pyrolysis, as presented in Table 2.

The results of determining the density and viscosity of the liquid pyrolysis products, obtained in accordance with the previously presented research methods, are presented in Table 4.

Table 5 shows the results of determining the fractional composition of liquid pyrolysis products, obtained in accordance with the described research methods.

The results presented in Table 4 and Table 5 indicate that the kinematic viscosity and density of the obtained pyrolysis products increase with increasing process temperature.

The average initial boiling point for the obtained fractions is 41 °C, and generally it decreases with increasing pyrolysis temperature. These results are associated with more active cracking reactions, which lead to the simultaneous production of lighter and heavier hydrocarbons. The average gasoline fraction content in the obtained liquid products is 60% by volume.

Since plastics, particularly polypropylene, are substances with very high molar masses and long hydrocarbon chains, during pyrolysis (thermal destruction in an oxygen-free environment), these chains break down, forming various hydrocarbons, including unsaturated ones. The results of determining the hydrocarbon group composition of the resulting liquids pyrolysis products are presented in Table 6.

Based on the data presented in Table 6, it can be seen that the predominant group of hydrocarbons in the obtained liquid pyrolysis products are olefins (monoolefins and diolefins), the highest content (78.07% by weight) of which is observed at a pyrolysis temperature of 500 °C (500 PP); the lowest content (72.55% by weight) of olefins is observed at a pyrolysis temperature of 450 °C (450 PP).

In addition, the pyrolysis products obtained at 450 °C (450 PP) contain the highest amount of normal paraffins (7.42% by weight). Based on the obtained results, the liquid pyrolysis product produced at a temperature of 450 °C (PP 450) was selected for the separation of the gasoline fraction and further studies. This product is characterized by a fairly high content of the gasoline fraction, average density and viscosity, the highest content of normal paraffins, and the lowest content of aromatic compounds.

### 4.2. Results of Determining the Characteristics and Composition of the Gasoline Fraction Separated from the Polypropylene Waste Pyrolysis Products

The results of determining the group hydrocarbon composition of the gasoline fraction separated from the polypropylene waste pyrolysis products (PP 450 sample), obtained by gas chromatography, are presented in Table 7.

From the results presented in Table 7, it can be seen that the predominant hydrocarbon groups in the separated gasoline fraction are olefins and aromatic hydrocarbons, and the total content of unsaturated compounds is 58.62% by weight. The content of these hydrocarbon groups is strictly regulated by state standards; for aromatic hydrocarbons, the limitation is 35% by volume, and for olefins, the limitation is 18% by volume [44]. Accordingly, it can be concluded that there is a need for hydrosaturation of this gasoline fraction for its further inclusion in the blending of commercial gasolines.

### 4.3. Identification of the Regularities of the Hydrosaturation Process Temperature Influence on the Composition of the Obtained Products

The results of determining the group hydrocarbon composition of the hydrosaturated gasoline fraction obtained under conditions of varying process temperature at a constant pressure of 20 bar, feedstock flow rate of 0.85 mL/min, and hydrogen flow rate of 35 mL/min are presented in Table 8.

As a result of the hydrosaturation carried out under conditions of varying process temperatures, the gasoline fraction separated from liquid products of polypropylene waste pyrolysis significantly changes its hydrocarbon composition. In general, the content of unsaturated hydrocarbons decreases during hydrosaturation, while saturated hydrocarbons, on the contrary, increase. It can be seen that as a result of hydrosaturation under conditions of varying process temperature, the olefin content in the gasoline fraction decreases relative to the feedstock by 11.990, 19.219, and 21.085% wt. at process temperatures of 300, 350, and 400 °C, respectively. The content of aromatic hydrocarbons decreases by 1.133, 12.465, and 7.953% wt. at process temperatures of 300, 350, and 400 °C, respectively. For benzene, an increase in the content relative to the feedstock of 0.499% wt. is observed at a process temperature of 300 °C in addition to a further decrease of 0.337 and 0.281% wt. at process temperatures of 350 and 400 °C, respectively. The content of n-paraffins increases with an increase in the process temperature from 300 to 400 °C relative to the feedstock by 23.001, 30.888 and 34.453% wt., and iso-paraffins decrease by 4.627, 2.010 and 0.858% wt., respectively. For naphthenes, a decrease in content of 5.180% wt. relative to the feedstock is observed at a process temperature of 300 °C, in addition to an increase of 3.715% wt. at a temperature of 350 °C and a decrease of 3.712% wt. at a temperature of 400 °C. A decrease in the content of oxygenates is also observed at all process temperatures relative to the feedstock.

The obtained trends are consistent with classical schemes for the conversion of various classes of hydrocarbons on aluminum–cobalt–molybdenum (ACM) catalysts in a hydrogen atmosphere [45].

Overall, during the hydrosaturation process, olefins and aromatic hydrocarbons are hydrogenated to saturated compounds, while naphthenic cyclostructures, after saturation, decyclize, become saturated, and transform into paraffins (up to and including cyclopentanes).

The maximum naphthene content and minimum aromatic hydrocarbon content at a process temperature of 350 °C, with a corresponding subsequent decrease and increase at a process temperature of 400 °C, are explained by an increase in the rate of hydrocracking reactions and a decrease in the rate of hydrogenation reactions with increasing temperature.

The decrease in isoparaffin content may be due to a shift in the equilibrium in isomerization reactions at relatively high temperatures toward normal paraffins. The increase in benzene content at a process temperature of 300 °C is due to the fact that alkyl chains in aromatic compounds begin to break off, but this temperature is insufficient for hydrosaturation of the benzene ring. During hydrosaturation, oxygen-containing compounds are converted to water and the corresponding hydrocarbons.

Therefore, the most favorable temperature for the hydrogenation process, from the standpoint of using the feedstock in gasoline production, is 350 °C, as the product obtained at these parameters is characterized by a relatively low olefin content and the lowest benzene content.

### 4.4. Identification the Regularities of the Feedstock Flow Rate in the Hydrosaturation Process on the Composition of the Obtained Products

The results of determining the group hydrocarbon composition of the hydrosaturated gasoline fraction obtained under conditions of a varying feedstock flow rate at a constant pressure of 20 bar, temperature of 350 °C, and hydrogen consumption of 35 mL/min are presented in Table 9.

As a result of hydrosaturation carried out under conditions of a varying feedstock flow rate, the gasoline fraction separated from the liquid products of polypropylene waste pyrolysis also significantly changes its hydrocarbon composition. It can be seen that as a result of hydrosaturation under conditions of a varying feedstock flow rate, the content of olefins in the composition of the obtained products decreases by 22.035, 19.219, and 17.763% wt. for a feedstock flow rate of 0.51, 0.85, and 1.19 mL/min, respectively. Aromatic hydrocarbons decrease by 10.823, 12.465, and 7.917% wt. for a feedstock flow rate of 0.51, 0.85, and 1.19 mL/min, respectively. For benzene, a decrease of 0.577 and 0.337% wt. is observed at a feedstock flow rate of 0.51 and 0.85 mL/min, and an increase of 0.230% wt. is observed at a feedstock flow rate of 1.19 mL/min. The content of n-paraffins increases by 40.888, 30.888 and 32.060% wt., and the content of iso-paraffins decreases by 2.729, 2.010 and 3.161% wt. for a feedstock flow rate of 0.51, 0.85 and 1.19 mL/min, respectively. For naphthenes, a decrease in content relative to the feedstock by 4.156% wt. is observed at a feedstock flow rate of 0.51 mL/min, and an increase of 3.715% wt. at a feedstock flow rate of 0.85 mL/min and a decrease of 2.881% wt. at a feedstock flow rate of 1.19 mL/min are observed. In addition, for all feedstock flow rates, a decrease in the content of oxygenates relative to the feedstock is observed.

As the feedstock flow rate increases, the olefin content of the products increases and the paraffin content decreases. The isoparaffin content also decreases slightly. It is worth noting that the naphthene content increases and the aromatic hydrocarbon content decreases in the feedstock flow rate range from 0.51 to 0.85 mL/min. With a further increase in the feedstock flow rate from 0.85 to 1.19 mL/min, the opposite trend is observed: the naphthene content begins to decrease, while the aromatic hydrocarbon content increases.

These trends are consistent with classical schemes for converting various classes of hydrocarbons on aluminum–cobalt–molybdenum (ACM) catalysts in a hydrogen atmosphere. As the feedstock flow rate increases, the degree of hydrogenation of unsaturated hydrocarbons decreases. This occurs because the contact time of the feedstock with the catalyst decreases, preventing the feedstock from becoming fully hydrosaturated [46]. Furthermore, based on this, it can be assumed that at a feedstock flow rate of 0.51 mL/min, the feedstock/catalyst contact time is sufficient to saturate the benzene ring and further convert naphthenes into paraffins (up to and including cyclopentanes). At a feedstock flow rate of 0.85 mL/min, the contact time is sufficient to saturate the benzene ring but insufficient for further conversion of naphthenes. At a feedstock flow rate of 1.19 mL/min, not all aromatic hydrocarbons have time to hydrosaturate and convert to naphthenes. A similar trend is observed for benzene: at a feedstock flow rate of 0.51 mL/min, a significant portion of the benzene is hydrosaturated. At a feedstock flow rate of 0.85 mL/min, the degree of hydrosaturation of benzene decreases due to insufficient contact time for the reactions to occur. At a feedstock flow rate of 1.19 mL/min, benzene does not have time to hydrogenate at all. Moreover, its content increases due to the formation of benzene due to the elimination of alkyl substituents from other aromatic hydrocarbons. Thus, the most preferable feedstock flow rate for the hydrosaturation process pressure in terms of use of the obtained product in gasoline production is 0.51 mL/min, as the product obtained under these parameters is characterized by the lowest olefin and benzene content.

### 4.5. Development of Commercial Motor Gasoline Recipes Using the Obtained Hydrosaturation Products

Table 10 presents the results of sample characteristic and composition calculations in «Compounding» software version 2.0 based on chromatographic analysis data.

According to the results presented in Table 10, sample 4HS is the most promising sample for inclusion in the blending of commercial motor gasolines in terms of hydrocarbon composition, as its olefin (18.75% vol.) and aromatic hydrocarbon (13.21% vol.) content, including benzene (0.87% vol.), is closest to the content standards for these hydrocarbon classes regulated by state standards. However, its characteristics require the inclusion of significant volumes of higher-octane components in the blending.

In turn, sample 1HS has the best performance characteristics of the presented ones: RON (72.9 points), density at 20 °C (730.9 kg/m^3^), and SVP (66.3 kPa). However, it contains 28.52% vol. olefins and 1.95% vol. benzene, which necessitates blending components that do not contain high levels of olefins and benzene when used in blending.

Using the Compounding software, blending recipes for Normal-80, Regular-92, Premium-95, and Super-98 gasolines brands were developed. The gasoline recipes were obtained using the following blending components: sample 1HS, sample 4HS, isomerizate (a product of the isomerization of the pentane–hexane fraction produced at the refinery), alkylate (a product of the alkylation of isobutane with olefins produced at the refinery), reformate (dynamic-bed catalyst technology), reformate (fixed-bed catalyst technology), straight-run gasoline, hydrotreated fluid catalytically cracking (HFCC) gasoline, and methyl tert-butyl ether (MTBE). The characteristics and group hydrocarbon composition of the standard blending components are shown in Table 11 and Table 12.

The resulting gasoline blending recipes are presented in Table 13.

The calculated characteristics of gasolines obtained according to the recipes proposed in Table 13 are presented in Table 14 and Table 15.

Based on the results presented in Table 13, Table 14 and Table 15, it can be concluded that samples 1HS and 4HS are promising components for inclusion in the blending of commercial gasolines, since all the gasolines obtained meet the requirements of the standards [44]; however, in order to increase the share of their inclusion in the production of commercial gasolines, it is possible to consider the option of further refinement in classical technologies used at oil refineries in order to further improve their characteristics.

## 5. Conclusions

1. The optimal parameters for the pyrolysis of polypropylene waste were determined. Of all the polypropylene waste pyrolysis modes presented, the 450 °C mode was found to be the most preferable for obtaining feedstock for subsequent hydrosaturation. The pyrolysis oil obtained at this temperature is characterized by a high gasoline fraction content (63% vol.) and a maximum normal paraffin content (7.42% wt.), which facilitates further processing. The physicochemical properties of the liquid pyrolysis products were also determined. It was established that with increasing pyrolysis temperature, the kinematic viscosity and density of the liquid products of polypropylene waste pyrolysis increase.
2. A hydrosaturation process for the gasoline fraction separated from the liquid products of polypropylene waste pyrolysis was implemented. It was determined how the hydrocarbon composition of the gasoline fraction changes under various process parameters of the hydrosaturation process on an Al-Co-Mo hydrotreating catalyst. It was found that increasing the hydrosaturation temperature from 300 to 400 °C reduced the olefin content to 19.76% by weight, but at 400 °C, an increase in the aromatic hydrocarbon content was observed. Increasing the feedstock flow rate from 0.51 to 1.19 mL/min reduced the hydrogenation depth due to a decrease in the feedstock–catalyst contact time.
3. Promising blending components were identified based on calculations in the “Compounding” software. Based on the obtained results, two samples, 1HS and 4HS, were identified as having the greatest potential for blending commercial gasolines in terms of hydrocarbon composition and operational characteristics. Sample 4HS (technological parameters: temperature of 350 °C and a feedstock flow rate of 0.51 mL/min) is the most preferable in terms of its composition, as it exhibits minimal olefin (18.7% vol.) and benzene (0.87% vol.) content but has a low octane number (RON 58.7). Sample 1HS (technological parameters: temperature of 300 °C and a feedstock flow rate of 0.85 mL/min) has higher octane characteristics (RON 72.9) and can be used as a high-octane component but requires blending with components that compensate for the increased olefin content.
4. Using the “Compounding” software, recipes were developed and the characteristics of Normal-80, Regular-92, Regular-95 and Super-98 gasolines were calculated using promising samples. It was shown that the resulting products can replace up to 35% by vol. (for Normal-80), up to 25% by volume (for Regular-92), up to 15% by volume (for Regular-95) and up to 5% by volume (for Super-98) of traditional components in the recipes of commercial gasoline, in full compliance with the requirements of the state standard for composition.

## Figures and Tables

**Table 1:** Technological parameters of the polypropylene waste pyrolysis process.

No.	Actual Feedstock Mass Rate, g/h	Reactor Temperature, °C	Pressure, MPa	Code of the Obtained Sample
1	591	410	1	410 PP
2	541	430		430 PP
3	547	450		450 PP
4	571	480		480 PP
5	534	500		500 PP

**Table 2:** Characteristics of using hydrosaturation catalyst.

Characteristic	Value
Nominal particle size, mm	1–2 × 1.4
Composition	Co/Mo on active Al oxide
Stoichiometric amount of sulfur, % mass.	11
Shape	Quatrefoil
Bulk density, kg/m^3^	37.0
Average length, mm	1.2
Mechanical strength, pounds/mm.	4.0
Abrasion loss, % wt.	2.0

**Table 3:** Technological parameters of catalytic hydrosaturation process.

No.	Reactor Temperature, °C	Feedstock Flow Rate, mL/min	Hydrogen F Low Rate, mL/min	Pressure, Bar	Yield of Target Product, % vol.	Code of the Obtained Sample
1	300	0.85	35	20	95.6	1HS
2	350	0.85			94.7	2HS
3	400	0.85			93.2	3HS
4	350	0.51			91.7	4HS
5	350	1.19			96.0	5HS

**Table 4:** Characteristics of the obtained pyrolysis oils.

Characteristic	Code of the Obtained Sample				
	410 PP	430 PP	450 PP	480 PP	500 PP
Kinematic viscosity at 20 °C, mm^2^/s	0.818	0.902	1.104	1.279	1.948
Density at 20 °C, g/cm^3^	0.731	0.740	0.747	0.754	0.755

**Table 5:** Fractional composition of the obtained pyrolysis oils.

Characteristic	Code of the Obtained Sample				
	410 PP	430 PP	450 PP	480 PP	500 PP
Initial boiling point (IBP), °C	44	46	41	36	38
End boiling point (EBP), °C	270	295	270	307	291
Fraction content IBP −180 °C, % vol.	71	56	63	53	56
Fraction content 180 °C—EBP, % vol.	21	38	27	35	27
Residue content, % vol.	8	6	10	12	17

**Table 6:** Group composition of obtained liquid pyrolysis products.

No.	Hydrocarbon Group	Content, % wt.				
		Code of the Obtained Sample				
		410 PP	430 PP	450 PP	480 PP	500 PP
1	N-paraffins	6.27	5.12	7.42	3.66	4.20
2	I-paraffins	5.25	3.22	4.35	2.20	2.14
3	Olefins	78.00	76.49	72.55	75.70	78.07
5	Naphthenes	2.83	2.24	1.88	1.95	1.86
6	Monoaromatic hydrocarbons	0.27	0.25	0.18	0.43	0.88
7	Unidentified components	7.38	12.68	13.63	16.06	12.85

**Table 7:** Group hydrocarbon composition of the gasoline fraction separated from the PP 450 sample.

No.	Hydrocarbon Group	Content, % wt.
1	N-paraffins	14.41
2	I-paraffins	15.90
3	Aromatic hydrocarbons	17.77
4	Naphthenes	10.50
5	Olefins	40.85
6	Oxygenates	0.58

**Table 8:** Group hydrocarbon composition of the hydrosaturated gasoline fraction obtained under conditions of varying process temperature.

Hydrocarbon Group	Content, % wt. at Process Temperature, °C			
	Feedstock	300	350	400
N-paraffins	14.407	37.408	45.295	48.860
I-paraffins	15.898	11.271	13.888	15.040
Aromatic hydrocarbons	16.321	15.188	3.856	8.368
including benzene	1.447	1.946	1.110	1.166
Naphthenes	10.500	5.320	14.215	6.788
Olefins	40.850	28.860	21.631	19.765
Oxygenates	0.577	0.007	0.005	0.014

**Table 9:** Group hydrocarbon composition of the hydrosaturated gasoline fraction obtained under conditions of a varying feedstock flow rate.

Hydrocarbon Group	Content, % wt. at Feedstock Flow Rate, mL/min			
	Feedstock	0.51	0.85	1.19
N-paraffins	14.407	55.295	45.295	46.467
I-paraffins	15.898	13.169	13.888	12.737
Aromatic hydrocarbons	16.321	5.498	3.856	8.404
including benzene	1.447	0.870	1.110	1.677
Naphthenes	10.500	6.344	14.215	7.619
Olefins	40.850	18.815	21.631	23.087
Oxygenates	0.577	0.008	0.005	0.008

**Table 10:** Results of sample characteristic and composition calculations.

Characteristic	Value					
	Feedstock	1HS	2HS	3HS	4HS	5HS
RON, points	96.9	72.9	64.2	59.4	58.7	63.7
MON, points	92.8	69.7	61.6	57.1	56.9	61.3
SVP, kPa	96.9	66.3	73.5	68.8	90.9	78.6
Density at 20 °C, kg/m^3^	737.6	730.9	722.2	720.7	714.2	715.0
Content, % vol.						
N-paraffins	13.96	35.81	42.18	46.57	50.98	45.23
I-paraffins	15.78	11.32	13.00	14.40	12.14	12.51
Naphthenes	10.08	4.13	12.30	5.32	4.92	6.31
Olefins	40.54	28.52	21.39	20.24	18.75	23.92
Aromatic hydrocarbons	19.08	20.21	11.12	13.46	13.21	12.02
including benzene	1.45	1.95	1.11	1.17	0.87	1.68

**Table 11:** Characteristics of standard blending components.

Standard Blending Components	Characteristic			
	Value			
	RON, Points	MON, Points	SVP, kPa	Density at 20 °C, kg/m^3^
Isomerizate т	89.8	87.9	62.8	656.4
Alkylate	96.7	94.2	34.7	695.0
Reformate fixed-bed catalytic technology	93.5	86.9	32.8	777.6
Reformate dynamic-bed catalytic technology	104.7	97.4	24.3	825.4
HFCC gasoline	91.6	85.7	43.2	737.7
Straight-run gasoline	51.2	47.5	17.6	699.4
MTBE	125.00	110.00	40.3	733.0

**Table 12:** Group hydrocarbon composition of standard blending components.

Standard Blending Components	Content, % vol.					
	N-Paraffins	I-Paraffins	Naphthenes	Olefins	Aromatic Hydrocarbons	Including Benzene
Isomerizate	0.54	93.83	5.49	0.00	0.06	0.03
Alkylate	5.12	94.88	0.00	0.00	0.00	0.00
Reformate, fixed-bed catalytic technology	10.36	24.54	1.62	0.00	64.54	4.19
Reformate, dynamic-bed catalytic technology	6.90	14.50	1.20	0.00	72.24	2.70
HFCC gasoline	4.45	31.36	13.41	26.87	23.77	0.72
Straight-run gasoline	22.97	61.18	13.98	1.44	0.48	0.03
MTBE	0	0	0	0	0	0

**Table 13:** Recipes for blending gasolines of different grades with the involvement of the obtained hydrosaturation products.

Blending Components	Content, % vol., for Gasoline Grades			
	Normal-80	Regular-92	Premium-95	Super-98
Using sample 1HS				
1HS	35	25	15	5
Alkylate	–	–	5	15
HFCC gasoline	25	30	45	45
Isomerizate	20	20	5	–
Reformate, dynamic-bed catalytic technology	–	10	15	20
MTBE	5	15	15	15
Straight-run gasoline	15	–	–	–
Using sample 4HS				
4HS	34	18	10	5
Alkylate	–	–	5	20
HFCC gasoline	40	49	45	35
Isomerizate	16	–	5	–
Reformate, dynamic-bed catalytic process	–	18	20	25
Reformate, fixed-bed catalytic process	10	–	–	–
MTBE	–	15	15	15

**Table 14:** Characteristics of gasolines obtained according to the proposed recipes, using sample 1HS.

Characteristic	Value			
	Normal-80, 1HS	Regular-92, 1HS	Premium-95, 1HS	Super-98, 1HS
RON, points	80.3	92.3	95.1	98.1
MON, points	76.0	86.4	88.4	91.2
SVP, kPa	51.2	50.6	44.0	38.9
Density at 20 °C, kg/m^3^	726.1	727.8	742.9	747.8
**Content, % vol.**				
N-paraffins	18.04	11.93	9.59	6.74
I-paraffins	41.84	35.08	29.73	34.25
Naphthenes	7.33	5.82	6.74	6.17
Olefins	17.53	15.94	17.68	14.71
Aromatic Hydrocarbons	10.41	16.66	21.23	22.98
including benzene	0.69	0.82	0.85	0.81

**Table 15:** Characteristics of gasolines obtained according to the proposed recipes, using sample 4HS.

Characteristic	Value			
	Normal-80, 4HS	Regular-92, 4HS	Premium-95, 4HS	Super-98, 4HS
RON, points	80.4	92.2	95.2	98.2
MON, points	76.4	85.4	88.5	91.5
SVP, kPa	61.5	48.2	44.6	39.0
Density at 20 °C, kg/m^3^	729.7	748.4	745.9	749.1
**Content, % vol.**				
N-paraffins	21.71	13.99	9.76	7.92
I-paraffins	36.60	22.01	30.16	36.56
Naphthenes	7.47	7.32	6.69	5.05
Olefins	17.89	17.94	15.12	11.58
Aromatic Hydrocarbons	16.35	23.60	23.17	23.93
including benzene	0.81	0.83	0.80	0.81

## Data Availability

Data are contained within the article. The data presented in this study are available on request from the corresponding author due to privacy concerns.
